# Functional Analysis of the Histidine N-Methyltransferase SETD3 in Endometriosis

**DOI:** 10.3390/ijms27136069

**Published:** 2026-07-07

**Authors:** Melanie Poloczek, Carolin Lisa Michaela Ludwig, Hanna Surmann, Theresa Strauß, Michael Gabriel, Matti Poutanen, Julia Oto, Ludwig Kiesel, Sebastian D. Schäfer, Lars Hanker, Joachim M. Weitzel, Martin Götte

**Affiliations:** 1 Department of Gynecology and Obstetrics, University Hospital Muenster, Albert-Schweitzer-Campus 1, 48149 Münster, Germany; m_polo04@uni-muenster.de (M.P.); hanna.surmann@uni-muenster.de (H.S.); t_stra12@uni-muenster.de (T.S.); juliaotomartinez@gmail.com (J.O.); ludwig.kiesel@ukmuenster.de (L.K.); seb.schaefer@alexianer.de (S.D.S.); lars.hanker@ukmuenster.de (L.H.); 2 Institute of Reproductive Biology, Research Institute for Farm Animal Biology (FBN), 18196 Dummerstorf, Germany; carolin.ludwig@psych.mpg.de; 3 Research Centre for Integrative Physiology and Pharmacology, Institute of Biomedicine, University of Turku, 20520 Turku, Finland; micawo@utu.fi (M.G.); matpou@utu.fi (M.P.); 4 Department of Obstetrics and Gyneacology, Institute of Medicine, University of Turku, 20014 Turku, Finland; 5 Haemostasis, Thrombosis, Arteriosclerosis and Vascular Biology Research Group, Medical Research Institute Hospital La Fe, 46026 Valencia, Spain; 6 Department of Gynecology and Obstetrics, Clemenshospital Münster, 48153 Münster, Germany; 7 Cells-in-Motion Interfaculty Centre (CiMIC), University of Münster, 48149 Münster, Germany

**Keywords:** SETD3, endometriosis, cytoskeleton, migration, invasive growth, infertility, actin methylation, in vitro study

## Abstract

Endometriosis is a disease associated with pain symptoms and reduced fertility, characterized by the presence of endometrial tissue outside the uterus. *SETD3* is an actin-specific histidine N-methyltransferase that regulates actin stability and flexibility. Here, we investigate the effects of altered *SETD3* expression on cytoskeletal function and endometriotic cell motility. *SETD3* expression in endometriotic lesions was analyzed using EndometDB, and in uteri of the superfertile Dummerstorf mouse line FL1 by RT-qPCR. The functional impact of siRNA-mediated *SETD3* depletion on endometriotic 12Z cells and primary endometriotic stroma cells was studied in vitro. Cell motility, contractility, invasiveness, morphology and gene expression were analyzed by RT-qPCR, Western blotting, immunofluorescence, scratch wound, collagen contraction and Matrigel invasion assays. In patient tissue, *SETD3* expression was slightly increased in deep endometriotic lesions, whereas *SETD3* was downregulated 1.6-fold in the uteri of superfertile mice. *SETD3* depletion delayed cell motility, reduced invasiveness of 12Z cells, and reduced the capability to contract collagen gels. Cytoskeletal gene expression was moderately changed. Our data suggest that the histidine N-methyltransferase *SETD3* contributes to cytoskeletal remodeling that plays a key role in cell migration and invasion. A dysregulation of *SETD3* could therefore be related to the pathogenesis of endometriosis, particularly in deep endometriosis.

## 1. Introduction

Endometriosis is a benign but invasive disease that affects women of reproductive age, resulting in a wide range of symptoms including severe pain and subfertility [1,2,3]. It has been demonstrated that endometriotic cells share cell biological characteristics with malignant tumors [4], in which case tumor cells are characterized by increased motility and migration, enabled by alterations of the cytoskeleton [5]. Hence, the cytoskeleton is likely involved in the pathogenesis of endometriosis, particularly deep endometriosis, promoting invasive cell behavior and the formation of ectopic lesions [6]. Indeed, previous work from our laboratory has shown that microRNA-dependent regulation of the actin bundling protein fascin and Neural Wiskott–Aldrich Syndrome protein *N-WASP/WASL*, a regulator of actin filament formation, regulates invasive growth of endometriotic cells [6]. Actin is the major protein of the cytoskeleton, essential for cell growth, division and migration [5,7]. Regulation of actin is extremely complex but indispensable for cytoskeletal integrity, and methylation is among the various mechanisms of actin regulation [7,8].

Post-translational methylation is one of the most common modifications in eukaryotic protein synthesis. Methylation of histones on arginine and lysine residues is well known, allowing for epigenetic regulation of gene expression [7]. However, methylation can also occur on non-histone proteins, which enables cell regulation apart from the DNA level. Methylation of β-actin was discovered in 1967 [9,10], but the enzyme responsible was only recently identified. The ubiquitously expressed protein *SETD3* (SET domain containing 3) has been reported as actin-specific histidine N-methyltransferase [7,11] *SETD3* catalyzes methylation of *β*-actin at His73 with the use of S-adenosyl methionine as methyl donor, leading to the stabilization of actin filaments [8,11].

Interestingly, *SETD3* knockdown animal models are still viable, and *SETD3* therefore does not impair the basal functions of actin [11,12]. Moreover, *SETD3* is described to have various protein interactions whose underlying pathways are still unknown. At least 172 *SETD3* interacting proteins have been identified but remained largely unstudied [13]. It is reported that *SETD3* is involved in the positive regulation of p53 [14], as well as in the upregulation of *VEGF* (Vascular endothelial growth factor) through methylation of *FoxM1* (Forkhead box protein M1) [13], the latter driving tumor proliferation and metastasis [15,16]. These properties indicate a potential role of *SETD3* in angiogenesis and oncogenesis, in accordance with altered *SETD3* expression levels in different carcinomas [17,18,19]. Liver cancer proliferation was enhanced by elevated *SETD3* levels as a result of inhibited degradation as *SETD3* itself is normally subject to a cell cycle-dependent downregulation by *FBXW7* (F-box/WD repeat-containing protein 7) and *GSK-3* (glycogen synthase-kinase 3) [20].

Due to these properties, we hypothesize that alterations in *SETD3* expression may affect cytoskeletal function and cell motility not only in malignant diseases but also in benign invasive diseases such as endometriosis. Furthermore, we state that our work is the first study on *SETD3* and endometriosis.

## 2. Results

### 2.1. Expression of SETD3 in Endometriosis Patient Tissue

**Figure 1:**
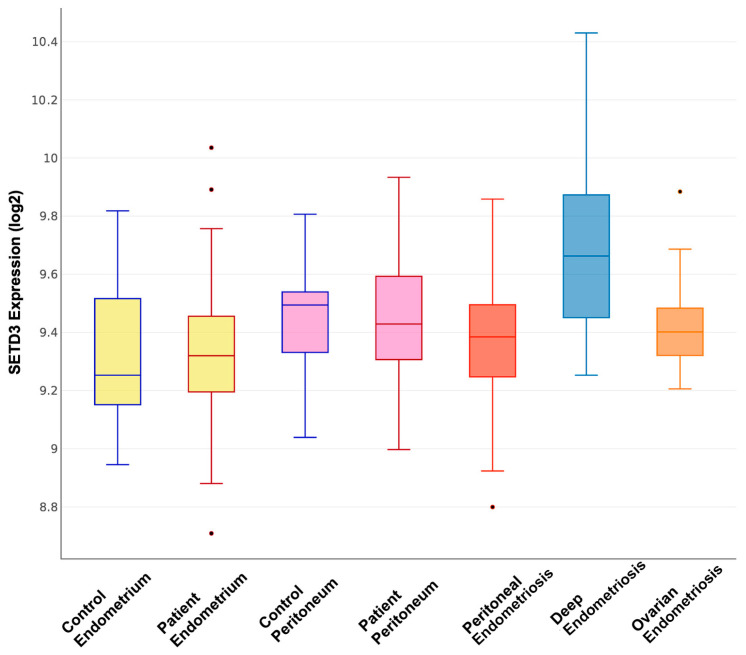
Analysis of *SETD3* expression in endometrial and endometriotic tissue. Normalized log2 SETD3 expression values from the Turku EndometDB were compared across control (*n* = 53) and patient (*n* = 115) endometrium, control and patient peritoneum, peritoneal lesions, deep infiltrating endometriotic lesions, and ovarian endometriotic lesions. Data are shown as box-and-whisker plots. The box plots display the distribution of the data, with the center line indicating the median, the box representing the interquartile range (IQR; 25th–75th percentile), and the whiskers extending to the minimum and maximum values. Outliers are displayed as individual points. Statistical comparisons were performed using an unpaired Student’s *t*-test. Statistical significance was defined as *p* < 0.05.

No studies so far have investigated the relevance of *SETD3* for endometriosis. Prior to analyzing *SETD3* function in vitro, we analyzed the mRNA expression of *SETD3* in 115 endometriosis patient tissues and 53 control tissues via EndometDB [21]. Analysis compared the expression in healthy control tissue to endometriotic tissue from differently localized lesions. Although data did not show any significant difference, the highest *SETD3* expression was found in deep infiltrating endometriosis tissue (Figure 1). This finding prompted us to investigate a possible functional role for SETD3 in endometriotic cells.

### 2.2. SETD3 Knockdown Affects Viability, Migration, Collagen Gel Contraction and Invasion of Endometriotic Cells

**Figure 2:**
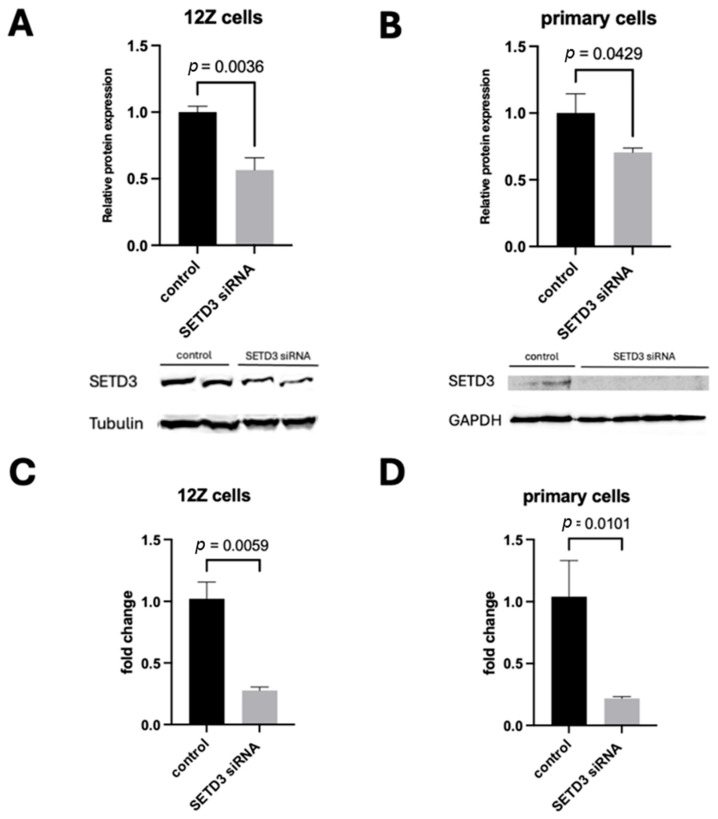
Efficient *SETD3* knockdown in 12Z cells and primary endometriotic stromal cells. Representative Western blots and densitometric quantification of *SETD3* protein expression following transfection with control siRNA or *SETD3*-targeting siRNA in 12Z cells (**A**) and primary endometriotic stromal cells (**B**). Protein expression was normalized to *Tubulin* or *GAPDH* and expressed relative to the control group. *SETD3* protein levels were significantly reduced following siRNA-mediated knockdown in both 12Z cells (*p* = 0.0036) and primary cells (*p* = 0.0429). Quantitative PCR analysis confirming efficient *SETD3* silencing in 12Z cells (**C**) and primary endometriotic stromal cells (**D**). Gene expression was normalized to *RPS18*. *SETD3* mRNA expression was significantly decreased following *SETD3* siRNA transfection in both 12Z cells (*p* = 0.0059) and primary cells (*p* = 0.0101). Data are presented as mean ± SEM.

**Figure 3:**
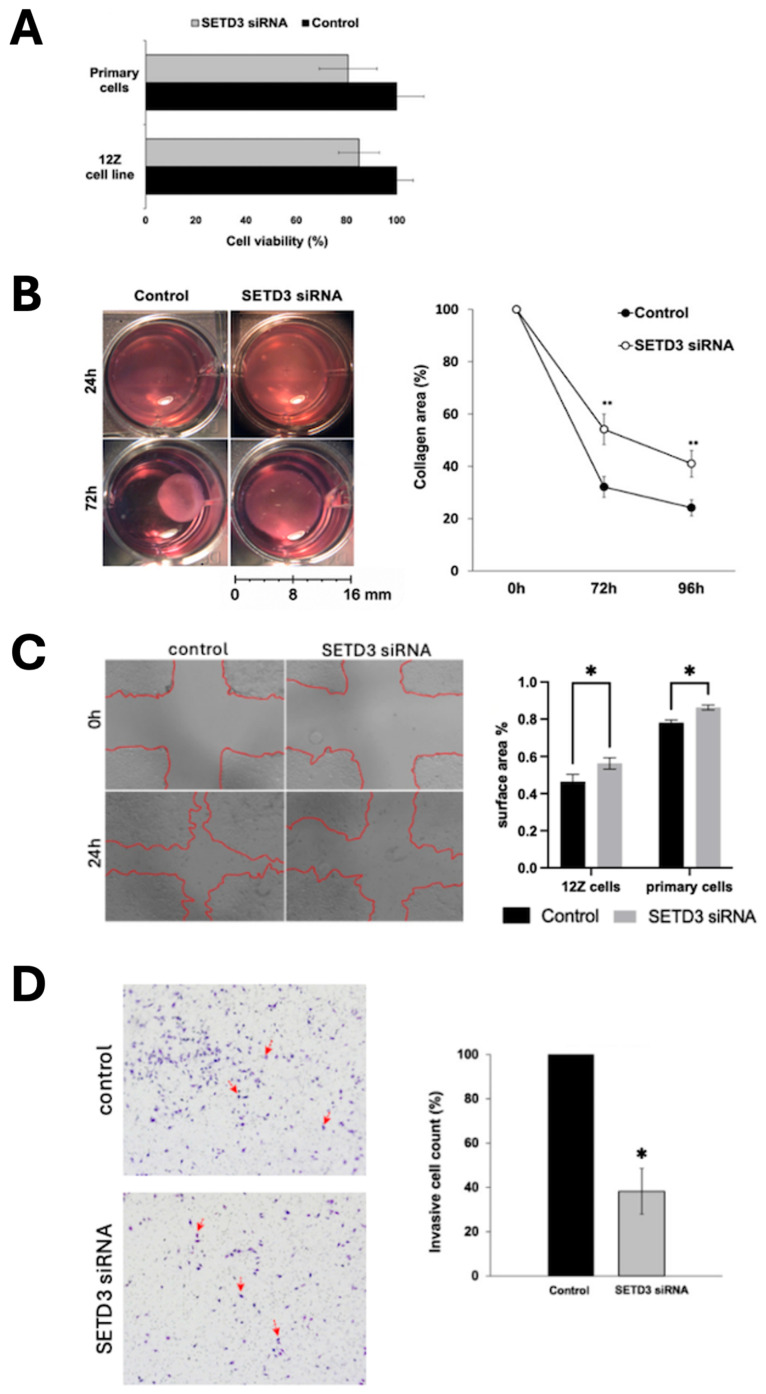
Functional analysis of the impact of *SETD3* knockdown in endometriotic cells. *SETD3* depletion leads to a decrease in cytoskeletal functions. Effects on cell viability as determined by MTT assay were not significant (**A**), with *p* = 0.133 for 12Z and *p* = 0.139 for primary cells of 4 patients), whereas collagen contraction of primary cells (one patient), migration of both cell types (four patients for primary cells) and Matrigel invasion of 12Z cells were significantly decreased (**B**–**D**). Left (**B**) and upper (**C**,**D**) panels show representative images of collagen contraction, scratch wound (12Z cells) and Matrigel invasion assays, respectively. Red arrows in panel (**D**) indicate invaded cells. The percent of contraction collagen area, cell-free surface area or the ratio of invasive cells were graphed and reveal a significant decrease in cell contractility, migration and invasiveness for *SETD3*-depleted cells. Data represent the mean ± SEM from three (invasion assay) or four (scratch, collagen, and MTT assays) independent experiments in duplicates. Bars or points with asterisks represent comparisons with statistically significant differences (*p* < 0.05).

We employed *SETD3* siRNA knockdown in vitro in the immortalized endometriotic cell line 12Z as well as in primary endometriotic stroma cells. Successful *SETD3* knockdown was confirmed by Western blot (Figure 2A,B) and RT-qPCR (Figure 2C,D). Following the downregulation of *SETD3*, no significant differences in the cell viability of 12Z and primary cells were noted (Figure 3A). However, we found significant restrictions in all other experiments with relevance for invasive cell behavior. The ability to contract collagen was significantly reduced in *SETD3*-depleted primary cells compared to control after 72 h (54.17% vs. 32.15%) and 96 h (41.03% vs. 24.16%) (Figure 3B). These findings were consistent with data obtained by the scratch wound assay, where both types of *SETD3* knockdown cells showed a significant delay in wound closure after 24 h compared to the control (59.4% vs. 46.5% for 12Z cells and 87.3% vs. 78% for primary cells) (Figure 3C). The Matrigel invasion assay revealed a significant reduction in invasiveness of *SETD3* siRNA treated 12Z cells by 72% with respect to control (Figure 3D).

### 2.3. SETD3 Knockdown Reduces β-Actin Methylation and Disrupts F-Actin Organization

**Figure 4:**
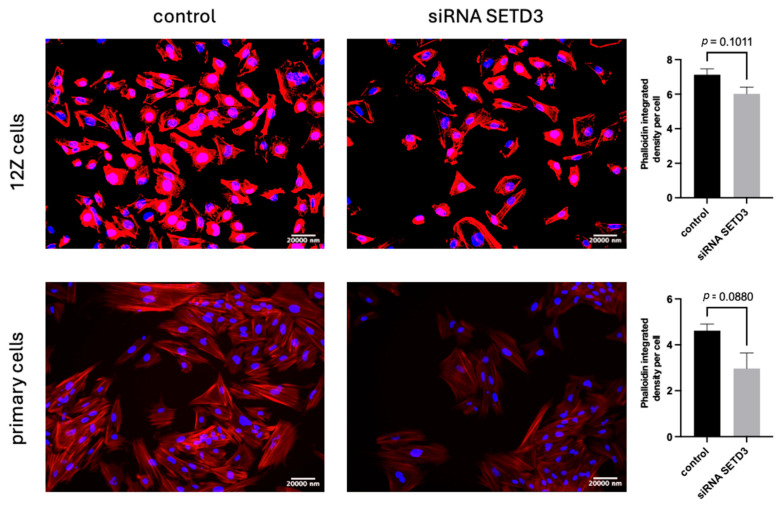
Representative immunofluorescence images of 12Z cells and primary endometriotic stromal cells transfected with control siRNA or siRNA targeting *SETD3*. *F-actin* was visualized by *Phalloidin* staining (red), and nuclei were counterstained with DAPI (blue). Quantification of *phalloidin* fluorescence intensity was performed using Fiji and normalized to the number of cells according to the DAPI staining. Data are presented as mean ± SEM. *SETD3* knockdown was associated with a reduction in *phalloidin* fluorescence intensity in both 12Z cells and primary endometriotic stromal cells. However, this difference did not reach statistical significance. Data are presented as mean ± SEM.

**Figure 5:**
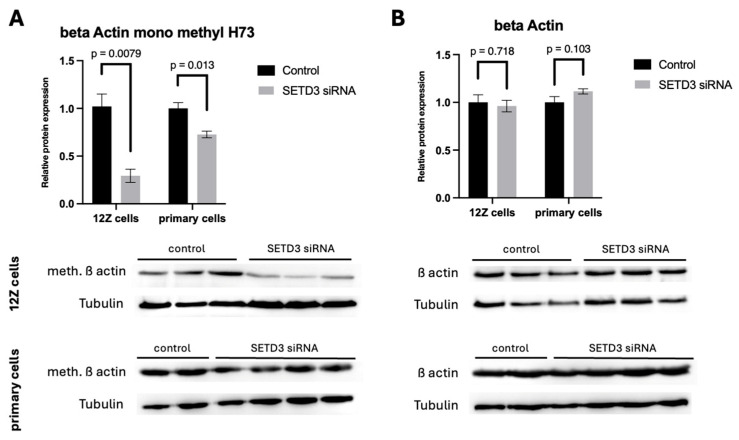
*SETD3* knockdown reduces β-actin histidine-73 *methylation* without affecting total β-actin protein levels in endometriotic cells. (**A**) Representative Western blots and densitometric quantification of histidine-73 methylated β-actin (meth. β-actin) in 12Z cells and primary endometriotic stromal cells transfected with control siRNA or *SETD3*-targeting siRNA. Methylated β-actin levels were normalized to Tubulin and expressed relative to the control group. *SETD3* knockdown significantly reduced *β-actin H73* methylation in both 12Z cells (*p* = 0.0079) and primary endometriotic stromal cells (*p* = 0.013). (**B**) Representative Western blots and densitometric quantification of total β-actin protein expression following *SETD3* silencing. Total *β-actin* protein levels were not significantly altered in either 12Z cells (*p* = 0.718) or primary endometriotic stromal cells (*p* = 0.103). Protein expression was normalized to tubulin and expressed relative to the control group. Data are presented as mean ± SEM.

Next, we performed fluorescence microscopy using phalloidin and DAPI staining (Figure 4). *SETD3* knockdown was associated with a reduction in phalloidin fluorescence intensity per cell compared with control cells; however, this difference did not reach statistical significance (7.121 ± 0.3437 vs. 6.015 ± 0.392, *p* = 0.1011 and 4.619 ± 0.2892 vs. 2.970 ± 0.6748, *p* = 0.0880). Actin stress fibers seemed to be less frequent in siRNA knockdown in primary cells. These findings support a possible role for *SETD3* in maintaining filamentous actin cytoskeletal architecture in endometriotic cells. Consistent with these findings, Western blot analysis revealed a significant reduction in *β-actin histidine-73* methylation (Figure 5A) following *SETD3* silencing in both 12Z cells (relative protein expression: 0.294 ± 0.069 vs. 0.9997 ± 0.115, *p* = 0.0079) and primary stromal cells (relative protein expression: 0.727 ± 0.035 vs. 1.0 ± 0.06, *p* = 0.013), whereas total *β-actin* protein levels remained unchanged (Figure 5B). Together, these results indicate that *SETD3* contributes to the maintenance of F-actin organization in endometriotic cells, likely through regulation of *β-actin* methylation rather than changes in total *β-actin* expression.

### 2.4. Impact of SETD3 Expression on the Expression of Genes with Relevance to Cytoskeletal Functions

**Figure 6:**
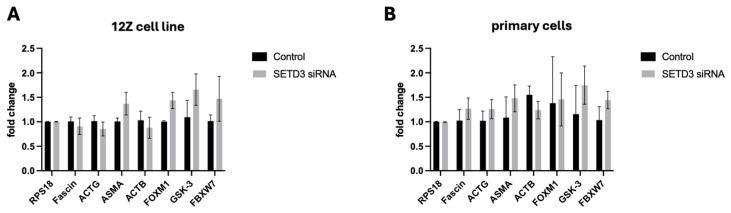
Quantitative RT-qPCR analysis of potential *SETD3* effector genes. Effect of *SETD3* downregulation on the expression of cytoskeleton-related genes. *Fascin*, *ACTG*, *ASMA*, *ACTB*, *FOXM1*, *FBXW7* and *GSK3* gene relative expression was quantified by RT-qPCR in 12Z cells (**A**) and primary endometrial stroma cells (**B**). Cells were transfected with control siRNA or *SETD3* siRNA. No significant alterations in the expression of the above-mentioned genes were detected. Individual experiments were normalized against RPS18 and relative expression was represented by 2^−ΔΔCt^. Data represent the mean ± SEM.

*Fascin*, *ACTB* (beta-actin), *ACTG* (gamma-actin), *ASMA* (alpha smooth muscle actin), *FOXM1* (Forkhead-Box protein M1), *FBXW7* (F-box/WD repeat-containing protein 7) and *GSK-3* (glycogen synthase kinase 3) are proteins with relevance for invasive diseases, some of them regulated by or regulating *SETD3* [11,13,20,21,22,23]. We performed RT-qPCR. Gene expression analysis revealed no significant alterations in 12Z cells or primary cells (Figure 6).

### 2.5. SETD3 Is Downregulated in the Uteri of the Dummerstorf Superfertile Mouse Line FL1

**Figure 7:**
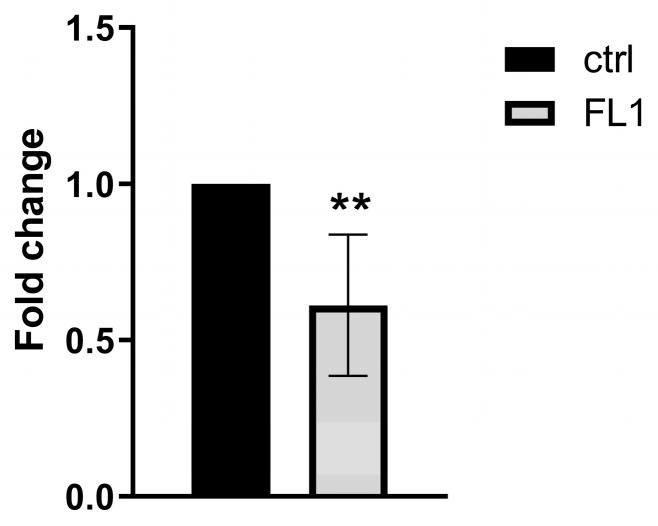
Quantitative RT-qPCR analysis of *SETD3* expression reveals significant downregulation in the uteri of Dummerstorf superfertile FL1 mice compared to normal controls. mRNA was isolated from the uteri of adult female FL1 and control mice during estrus, converted to cDNA, and subjected to qPCR analysis for *SETD3*. 36B4, RPS18 and B2m served as reference genes. ** *p* < 0.01, control *n* = 9; FL1 *n* = 10, error bars = SD. Control samples served as calibrators and were therefore set to 1.

Apart from pain, subfertility is associated with endometriosis [2,3]. To investigate a possible association of *SETD3* with fertility, we analyzed its expression in the uteri of a unique mouse model associated with superfertility, the Dummerstorf superfertile mouse line FL1. This mouse line 1 was selected for increased litter size and the total birth weight of the litters for almost 200 generations [24,25]. During the selection process FL1 mice almost doubled the number of pups per litter, as well as the total birth weight of the entire litters compared to the unselected control line (ctrl) in the first parturition and show no signs of growth retardation in the offspring [26]. FL1 females ovulate approximately twice as many oocytes compared to ctrl mice [27]. Furthermore, not only do FL1 mice deliver a high number of pups in the first pregnancy, they are able to deliver large litter sizes with a high reproductive mating rate over a long-time period without health issues [28] and therefore are a worldwide unique animal model for increased female reproductive performance, high fertility and longevity. To analyze *SETD3* expression, uteri of 10 FL1 and 9 control mice were collected during estrus and snap-frozen, followed by RNA isolation, reverse transcription and qPCR analysis for *SETD3* expression. A significant, 1.6-fold downregulation of *SETD3* was observed in the uteri of superfertile mice compared to controls (Figure 7), indicating a possible connection of *SETD3* expression to fertility in female mice.

## 3. Discussion

*SETD3* has been identified to be an actin-specific histidine N-methyltransferase [7,11]. Structural analysis has indicated that methylation regulates actin’s flexibility and stability [8], and the relevance of *SETD3* has been studied for various cancer types [29,30]. These properties make *SETD3* a promising candidate to participate in the development of endometriosis.

Altered expression levels of *SETD3* have been found in different cancer cells; it is reported that aberrant *SETD3* levels promote tumor proliferation in hepatocellular carcinoma, in line with expressional changes of *SETD3* described for renal, ovarian and breast cancer in relation to altered patient survival [17,18,19,30]. Gene expression analysis of *SETD3* is useful to evaluating its clinical potential as biomarker and to obtaining initial data on its relevance for endometriosis. Although *SETD3* expression was not significantly different between endometriosis subtypes in this study, a trend towards higher expression in deep-infiltrating lesions was observed. Interestingly, our functional experiments demonstrated that *SETD3* contributes to migration, invasion, and collagen contraction, processes that are characteristic of the invasive phenotype of deep endometriosis. As a specific substrate of *SETD3*, β-actin is methylated and thus stabilized under physiological conditions [7]. Following the downregulation of *SETD3*, we assumed that the stabilization of actin filaments was no longer applicable, which may have led to a decrease in cytoskeletal stability. We were able to demonstrate a significant reduction in *histidine-73 methylated β-actin* following *SETD3* knockdown in both 12Z cells and primary endometriotic stromal cells, whereas total β-actin protein levels remained unchanged. These findings confirm that *SETD3* depletion impairs β-actin methylation rather than overall β-actin expression and provide mechanistic evidence linking SETD3 activity to cytoskeletal regulation in endometriotic cells. Expression of unmethylated actin isoforms, *ACTB*, *ACTG* and *ASMA*, was not altered in our study while SETD3 is described to potentially methylate all actin isoforms in vitro [11].

Strong results for the effect of *SETD3* depletion on cytoskeletal function were observed in our functional experiments. Endometriotic cells lacking *SETD3* showed decreased cell motility, collagen contractility and invasiveness. In agreement with that, the knockdown of SETD3 in breast cancer cells markedly reduced invasiveness and collagen contraction ability [30]. These findings indicate that reduced *SETD3* expression could affect cytoskeletal function not only in malignant but also in benign invasive diseases, more precisely in endometriosis. Cell viability was not significantly decreased in our cells, but *SETD3* deprivation is known to reduce cell proliferation in breast cancer and liver cancer cells [20,30]. Other studies revealed reduced cell viability in accordance with *SETD3* overexpression, characterizing *SETD3* as an apoptotic regulator [31]. *SETD3* initiates apoptosis by the positive regulation of p53, which characterizes *SETD3* as a bivalent regulator of cell viability depending on the particular entity [14]. For endometriosis, various studies reported a reduction in apoptosis during menstruation which facilitates the formation of ectopic lesions [32,33]. Therefore, potential apoptotic effects of *SETD3* in endometriosis are worth addressing in future studies.

Interestingly, most of *SETD3* protein is localized in the cytoplasm of the cell [12,20]. To further investigate the consequences of impaired β-actin methylation on cytoskeletal organization, we performed *phalloidin* staining to visualize filamentous actin. *SETD3* knockdown resulted in reduced *phalloidin* fluorescence intensity in both 12Z cells and primary endometriotic stromal cells. Importantly, these alterations occurred despite unchanged total *β-actin* protein levels, suggesting that *SETD3* primarily affects the functional state and organization of filamentous actin rather than its overall abundance. Together with the observed reduction in β-actin H73 methylation, these findings support a model in which *SETD3*-dependent actin methylation contributes to the maintenance of F-actin architecture and cytoskeletal dynamics required for cell migration, invasion, and collagen contraction. It is known that *SETD3* is not necessary for basal actin function as *SETD3* knockdown animals are still viable [11,12]. Downregulation of *SETD3* may therefore not affect basal actin morphology in endometriotic cells but may result in reduced actin methylation potentially modifying the functional status of actin and, subsequently, in reduced cell motility, contractility and invasiveness.

To assess a potential link between *SETD3* and fertility, we employed a unique and informative resource, the Dummerstorf superfertile mouse model FL1 [24,25,26,27]. In line with our in vitro and clinicopathological investigation, *SETD3* expression was significantly downregulated in FL1 mice compared to normal controls. If applied to the situation in humans, in an endometriosis context, a low expression of *SETD3* in uterine tissue (specifically the endometrium) could reduce the occurrence of endometriosis by hampering invasive growth and therefore establishment of the endometriotic lesion when distributed via classical etiological routes such as retrograde menstruation. A reduction in endometriotic lesions would in turn reduce a number of pathogenetic mechanisms thought to be involved in endometriosis-associated infertility, including an enhanced local inflammatory response, which may indirectly affect the ovarian reserve, and anatomical distortions due to adhesions and fibrosis [1,2].

Interestingly, although the expression of *SETD3* in the uterus of FL1 mice is decreased, it has been shown that *SETD3* mRNA expression is significantly increased in ovaries [26] and in granulosa cells of antral follicles (Ludwig CLM et al. unpublished) of FL1 mice. Thus, while decreased mRNA levels of *SETD3* in the uterus potentially prevent invasive growth of endometriotic tissue, increased *SETD3* levels might inhibit apoptotic pathways in the ovary and in granulosa cells, which in turn leads to increased follicular survival, improved follicular development and therefore higher ovulation rates in FL1 mice. However, it should be taken into account that the high *SETD3*/low apoptotic ovarian phenotype of FL1 mice is not associated with any negative health issues.

Finally, *SETD3* may also affect fertility at very late stages of pregnancy as *SETD3*-deficient female mice have severely decreased litter sizes due to primary maternal dystocia [11]. However, we consider a complete knockout of *SETD3* in humans a rare event, whereas the more moderate regulation observed in the Dummerstorf superfertile FL1 mouse appears to be closer to a physiological regulation.

Our study demonstrated that *SETD3* depletion reduces β-actin histidine methylation and is accompanied by impaired F-actin organization, decreased migration, invasion, and collagen contraction. Together, these findings identify *SETD3*-mediated β-actin methylation as an important regulator of cytoskeletal dynamics in endometriotic cells and suggest that *SETD3* may contribute to the invasive phenotype characteristic of endometriosis.

## 4. Materials and Methods

### 4.1. SETD3 Gene Expression Analysis in Patient Tissues

Examination of *SETD3* gene expression in endometriosis patients’ tissue and controls was performed via Turku Endometriosis database EndometDB (https://endometdb.utu.fi/, accessed on 30 October 2022). This web-based database incorporates the expression data from 115 patients and 53 controls as described previously [21]. All clinical and sample data were selected for analysis and *SETD3* expression data was presented in boxplots on a log2-scale.

### 4.2. Material

Fetal calf serum (FCS) was from PAN-Biotech (Aidenbach, Germany). Unless stated otherwise, all chemicals were from Sigma-Aldrich (St. Louis, MI, USA).

### 4.3. Cell Culture

The immortalized epithelial endometriotic human cell line 12Z was kindly provided by Prof. Anna Starzinski-Powitz, Frankfurt, Germany [34]. Primary endometriotic stroma cells were obtained from biopsies of six women with endometriosis who underwent surgical treatment at the Department of Gynecology of Münster University Hospital. Patient characteristics are shown in Table 1. Primary cells were isolated as described previously [35]. Cells were cultured in DMEM supplemented with 10% FCS and 1% penicillin-streptomycin in a humidified atmosphere of 7.5% CO_2_ at 37 °C. The use of 7.5% CO_2_ is consistent with the bicarbonate buffering system of the medium and ensures maintenance of physiological pH during cell culture.

### 4.4. siRNA Transfection

A total of 200,000 cells/well of a six-well plate were cultured in DMEM for 24 h. siRNA transfection was performed using Dharmafect reagent (Dharmacon™, Lafayette, LA, USA) according to the supplier’s protocols. This reagent contained 840 µL Opti-MEM™ (Thermo Fisher Scientific, Waltham, MA, USA), 80 µL 20 nM *SETD3* siRNA/Opti-MEM™ (Silencer™ Select Pre-Designed siRNA (ID s38639), Thermo Fisher Scientific, Waltham, MA, USA) or negative control siRNA/Opti-MEM™ (Silencer™ Select Negative Control No. 1 siRNA (Cat. no. 4390844), Thermo Fisher Scientific, Waltham, MA, USA), and 80 µL 2.5% Dharmafect/Opti-MEM™ solution in a total volume of 1 mL. Cells were incubated for 24 h at 37 °C and 7.5% CO_2_. The medium was then changed to DMEM with FCS. mRNA and protein extraction were performed 48 h after transfection.

### 4.5. Quantitative Real-Time PCR of Endometriotic Cell Models

mRNA isolation was performed with an InnuPREP RNA mini kit (Analytikjena, Jena, Germany). The mRNA concentration and purity were assessed using an Eppendorf BioPhotometer (Eppendorf, Hamburg, Germany). mRNA was then transcribed into cDNA using the cDNA Reverse Transcription Kit (Applied Biosystems, Foster City, CA, USA) according to the supplier’s instructions. For cDNA synthesis 1000 ng of RNA was used for 12Z cells, and 500 ng of RNA was used for primary cells. Quantitative real-time PCR was performed in duplicates for each target gene using Takyon™ ROX SYBR^®^ MasterMix blue dTTP (Eurogentec, Liège, Belgium), and gene expression levels were measured via a 7300 Real-Time PCR detection system (Applied Biosystems, Foster City, CA, USA). Transcriptional analysis was performed using the comparative cycle threshold method and *RPS18* was used as a housekeeping gene. Primer sequences are listed in Table 2.

### 4.6. Western Blot

Cell lysates were prepared with a mixture of 222 µL Blue Loading Buffer (Cell Signaling, Cambridge, UK), 22.2 µL 30× Reducing Agent (Cell Signaling, Cambridge, UK) and 422 µL H_2_O for a six-well plate. Then, 15 µL of protein per lane was separated on 10% SDS gels and transferred to nitrocellulose membranes (GE Healthcare, Marlborough, MA, USA). After one hour of blocking with 5% skimmed milk powder in 1× TBST buffer, detection was performed with *SETD3* rabbit polyclonal primary antibody (1:1000, Sigma-Aldrich, Darmstadt, Germany, cat. no. HPA003639), *β-actin H73 methylation* rabbit monoclonal primary antibody (1:1000, Abcam, cat. no. ERP29292-70), *β-actin* rabbit polyclonal primary antibody (1:1000, Cell Signaling Technology, cat. no. 4967) and anti-rabbit secondary antibody (1:10,000, Sigma-Aldrich, cat. no. 401353). Membranes were subjected to a chemiluminescence reaction, and membranes were stripped with 0.2 M glycine buffer (pH 2.5) afterwards. For tubulin detection as loading control, membranes were subjected to the procedure described above using *tubulin* mouse antibody (1:10,000, Sigma cat. no. T5168) and anti-mouse secondary antibody (1:10,000, Sigma-Aldrich, cat. no. 401253). For *GAPDH* detection as loading control, *GAPDH* mouse antibody (1:1000, Gene Tex, Irvine, CA, USA, cat no. GTX627408) and anti-mouse secondary antibody (1:10,000, Sigma-Aldrich, cat. no. 401253) were used.

### 4.7. MTT Proliferation Assay

Cell viability was determined after treatment with control or *SETD3* siRNA by the MTT (methylthiazolyldiphenyltetrazolium bromide) assay. *SETD3*-depleted or control cells (4 000 maximum) were plated in 96-well plates with DMEM medium without phenol red (Gibco^®^, Thermo Fisher Scientific, Waltham, MA, USA) with FCS and incubated for 96 h at 37 °C. Then, the medium was changed to 20 µL of MTT solution (250 mg MTT powder, Sigma-Aldrich, cat. no. 5655, in 50 mL PBS) per well. After 4 h of incubation, 100 µL of a stopping solution (50% N,N-Dimethylformamid and 10% SDS in H_2_O) per well was applied. Photometric analysis was performed after a 24 h incubation period at room temperature in a VersaMax^®^ ELISA Microplate Reader (Molecular Devices, San José, CA, USA) at a wavelength of 595 nm. The MTT assay was performed with 12Z cells and primary cells from four patients.

### 4.8. Scratch Wound Assay

*SETD3* knockdown cells and controls were cultivated in DMEM with FCS in six-well plates in duplicates. Adherent cells were washed with PBS and a cross-shaped scratch was performed using a sterile 1000 µL pipette tip. The media was changed to remove detached cells, followed by a 24 h incubation at 37 °C. Cell migration into the free surface area was determined at time intervals of 0, 5, 10, and 24 h after the initial scratch. Images of the surface areas were taken under an Axiovert 100 microscope using the Axio Vision program (Carl Zeiss, Jena, Germany; https://www.micro-shop.zeiss.com/en/us/, accessed on 1 March 2026). The ratio of cell migration was calculated as the percentage of the free surface cross-area at 24 h compared with the area of the initial scratch at 0 h. ImageJ 1.x software (National Institutes of Health, Bethesda, MD, USA) was used for surface area measurement. The scratch wound assay was performed with 12Z cells and primary cells from four patients.

### 4.9. Collagen Contraction Assay

*SETD3*-depleted primary cells or controls were diluted to 400,000/300 µL DMEM. Then, collagen gels were prepared on ice using 714 µL collagen type I (4.88 mg/mL, Corning, cat. no. 354236, Corning, NY, USA), 16.4 µL NaOH, 169 µL H_2_O, and 100 µL 10X PBS for each SETD3-depleted cells and controls. A total of 250 µL of the respective cell suspension was mixed with the collagen gel to create cell-embedded collagen gels with a cell–collagen ratio of 1:4. The gels were transferred into a 24-well plate (500 µL/well) and incubated at 37 °C for 1 h to allow polymerization. Then, 1 mL of DMEM with FCS was added to each well and incubated at 37 °C. Photographs were taken at 24 h intervals using a digital camera (resolution of 3024 × 4032 pixels) and an Axiovert 100 microscope (Carl Zeiss, Jena, Germany) to monitor the contraction progress. The collagen surface areas of the gels were measured using the ImageJ 1.x program (National Institutes of Health, Bethesda, MD, USA). As the assay required high cell numbers, it was performed with stroma cells of a single patient (OP23) only.

### 4.10. Matrigel Invasion Assay

*SETD3*-depleted 12Z cells and controls were diluted to 100,000 cells/2 mL in DMEM with FCS. Then, 500 µL (25,000 cells) were transferred to Matrigel chambers (Corning^®^ BioCoat™ Matrigel^®^ Invasion Chambers, cat. no. 354480, Corning, NY, USA) and incubated for 24 h at 37 °C. Subsequently, the media was carefully replaced by FCS-free DMEM and invasion was initiated by filling the Matrigel wells with 750 µL of DMEM with FCS. After another 24 h incubation period, the media were removed, and cells were fixed in methanol and washed with PBS. The cells were then stained in 1% toluidine blue in SB buffer (Sigma, cat. No. T3260) for six minutes and washed with H_2_O. For each membrane, cells in two visual fields were counted under an Axiovert 100 microscope (Zeiss, Jena, Germany; magnification 10×). The Matrigel invasion assay was performed with 12Z cells.

### 4.11. Fluorescence Microscopy

*SETD3*-depleted 12Z cells and controls were placed in a 96-well plate. Seeding densities were optimized for each cell type to achieve comparable confluency and imaging quality at the time of analysis (10,000 cells each for 12Z cells and 7500 cells each for primary cells from one patient). All media were removed, and cells were fixed in a 4% formaldehyde solution (100 µL/well) for 10 min, then washed with 1 mL PBS for 5 min and incubated in 0.1% Triton (500 µL/well; Sigma-Aldrich, USA) for 10 min. Washing was repeated, followed by a 25 min incubation in 1% BSA/PBS (Thermo Fisher Scientific, Waltham, MA, USA). Actin filaments were stained with *Phalloidin* CruzFluor™ (1:1000, Santa Cruz Biotechnology, Dallas, NY, USA) in blocking solution (1% BSA, 10% FCS, 0.02% Triton) for 60 min. Washing was repeated before cells were stained with DAPI (1 µL in 25 mL PBS) for 10 min. Washing was repeated. Cells were then conserved in 100 µL PBS/well and examined by immunofluorescence microscopy on a Keyence BZ-X1000 Series Microscope (Keyence Corporation, Osaka, Japan). For immunofluorescence quantification, the integrated fluorescence intensity was measured and normalized to the number of DAPI-positive nuclei in the corresponding field of view.

### 4.12. Uterine SETD3 Expression Analysis in the Dummerstorf Superfertile Mouse (FL1)

#### 4.12.1. Animals and Housing

All procedures were performed following national and international guidelines and approved by the federal state of Mecklenburg Western-Pommerania, Germany (approval no. AZ 7721.3-2-015/20).

Female mice, bred at the Research Institute for Farm Animal Biology (FBN), Dummerstorf, Germany, were housed in groups of three animals per cage. The FL1 line, a mouse line selected for high fertility, was compared to non-selected control line, as described in references [27,36,37,38]. A commercial breeding diet for rodents and water was provided ad libitum, and illumination of animal facilities was between 6.00 a.m. and 6.00 p.m. A male mouse was kept for acoustic, visual and olfactory stimulus in a separate cage.

#### 4.12.2. Sample Procedure

To avoid cycle-related alterations of gene expression, samples were taken exclusively in estrus. Vaginal cytology was used to determine the stage of the estrous cycle like previously described [27,36,37]. Mice at the age of 9–13 weeks were euthanized by nitrogen inhalation. After determination of death, samples were taken. The uterus was cleaned from fat tissue and immediately snap-frozen and stored at −70 °C.

#### 4.12.3. RT-qPCR

Samples were pulverized in liquid nitrogen. RNA was extracted with the RNeasy Plus Mini Kit (Qiagen, Hilden, Germany), and RNA quality was assessed using the A260/A280 ratio. Only samples with ratios between 2.0 and 2.1 were included for further analyses. cDNA was synthesized from 400 ng of total RNA using an iScript cDNA Synthesis Kit (Biorad, München, Germany) according to the manufacturers protocol. Primers were designed using Primer-BLAST (https://tib-molbiol.com/, accessed on 1 March 2026) and purchased from TIB Molbiol (Berlin, Germany). Samples were analyzed in duplicates using 4 µL primer mix, 5 µL SsoAdvancedTM Universal SYBR Green Supermix (Biorad, München, Germany) and 1 µL of cDNA reaction solution and was loaded onto a 96-well plate and amplified by real-time PCR (iCycler, Biorad, München, Germany). RT-qPCR was performed using the following cycling conditions: initial denaturation at 94 °C, followed by 40 cycles of 94 °C for 10 s, 60 °C for 30 s, and 72 °C for 45 s. Amplification specificity was verified by melt curve analysis. Only reactions exhibiting a single distinct melting peak were considered specific and included in the analysis. The results of the mRNA abundance are calculated relative to a combination of the reference genes *36B4*, *RPS18* and *B2m*. Relative gene expression and statistical analysis was calculated using the Relative expression software tool, version REST 2009 [38,39]. Control samples served as calibrators and were therefore set to 1. Primer sequences are listed in Table 3.

### 4.13. Statistical Analysis

The data were tested for significance employing Student’s unpaired *t*-test. RT-qPCR data in the mouse model were analyzed using REST (Relative Expression Software Tool), version REST 2009 [38,39]. All experimental assays were repeated at least three times in duplicates. Data are presented as the mean values ± SEM or SD (animal model). Significant *p*-values are indicated as follows: *p* < 0.05 by one asterisk *, *p* < 0.01 by two asterisks ** and *p* < 0.001 by three asterisks ***.

### 4.14. Ethical Approval of Primary Cell Work

This study was approved by the local ethics committee (Ethikkommission der Ärztekammer Westfalen-Lippe und der Medizinischen Fakultät der Westfälischen Wilhelms-Universität Münster; reference no. 1 IX Greb from 19 September 2001 and updated on 6 December 2012) and reference number 2025-693-f-S (2025).

No generative artificial intelligence (GenAI) has been used in this paper.

## Figures and Tables

**Table 1:** Characteristics of endometriotic biopsies (adapted from Ramirez Williams et al. [35]).

Laboratory Code	Patient Age at Biopsy	Location of Biopsy	rASRM Score	Endometriosis Manifestations
OP8	33	Pouch of Douglas/ Sacrouterine ligament	II	Pelvic wall Recessus ovarii (left) Vesicouterine pouch Sacrouterine ligaments Pouch of Douglas
OP23	45	Periureteral	IV	Urinary bladder Peritoneum Vagina Pouch of Douglas Deep infiltrating: rectum, ovaries Disseminated: peritoneum
OP26	35	Pelvic wall	III	Gut Left ovary Rectovaginal septum Pouch of Douglas Vagina Peritoneal (pelvic) Deep infiltrating: Sacrouterine ligaments, pelvic wall
OP28	39	Peritoneum	III	Peritoneum, urinary bladder (disseminated, deep infiltrating), Pelvic wall near ureter (disseminated)
OP5	35	Vagina	III	Septum rectovaginale, DIE, vagina, uterus, rectum
OP10	19	Pelvic wall	II	Periurethral, pelvic wall, septum rectovaginale, vagina

rASRM: revised American Society for Reproductive Medicine.

**Table 2:** Primer sequences for RT-qPCR.

Gene	Sequence (5′–3′)
*Fascin*	fwd: GGAGACCGACCAGGAGAC
	rev: CATTGGACGCCCTCAGTG
*ACTG*	fwd: ATGGAAGGAAACACGGCTC
	rev: CACTCTGTTCTTCCGCCG
*FOXM1*	fwd: ATACGTGGATTGAGGACCACT
	rev: TCCAATGTCAAGTAGCGGTTG
*RPS18*	fwd: GCAGAATCCACGCCAGTACAAG
	rev: GCTTGTTGTCCAGACCATTGGC
*ACTB*	fwd: TCAAGATCATTGCTCCTCCTGAG
	rev: ACATCTGCTGGAAGGTGGACA
*ASMA*	fwd: ACTGAGCGTGGCTATTCCTCCGTT
	rev: GCAGTGGCCATCTCATTTTCA
*FBXW7*	fwd: AGTACCACTGGGCTTGTACC
	rev: CTCTGGTCCACTCCAGCTCT
*SETD3*	fwd: CAACCTGGAAGATGACCGCTGT
	rev: CACTGTGGATCACAAACTCTGCG
*GSK-3*	fwd: GGAGAACTGGTCGCCATCAAG
	rev: ACATTGGGTTCTCCTCGGACC

fwd: forward, rev: reverse.

**Table 3:** Primers used for murine gene expression analysis.

Gene	Sequence (5′–3′)
*SRTD3*	fwd: TGTCTGTTACTTTTGATGGGAAAA
	rev: AGAATTCGGTCTTGAGAATACAGG
*RPS18*	fwd: ACCATCATGCAGAACCCACGACAGT
	rev: CAGGTCCTCACGCAGCTTGTTGTCT
*36B4*	fwd: AAGCGCGTCCTGGCATTGTCT
	rev: CCGCAGGGGCAGCAGTGGT
*B2M*	fwd: TTCTGGTGCTTGTCTCACTGAC
	rev: GCAGTTCAGTATGTTCGGCTTC

fwd: forward, rev: reverse.

## Data Availability

Patient gene expression datasets were retrieved and analyzed using the publicly accessible EN-DOMET Turku Endometriosis Database (https://endometdb.utu.fi/, accessed on 22 April 2022) [21]. All experimental data presented in this study are contained in this manuscript. Original data supporting the findings of this study are available upon request from the corresponding authors.

## Abbreviations

The following abbreviations are used in this manuscript:

FBXQ7	F-box/WD repeat-containing protein 7
FOX M1	Forkhead box protein M1
GSK-3	Glycogen synthase-kinase 3
RT-qPCR	Reverse transcriptase quantitative real-time polymerase chain reaction
SETD3	SET domain containing 3
NWASP	Neural Wiskott–Aldrich Syndrome protein
VEGF	Vascular endothelial growth factor

## References

[1] Zondervan K.T., Becker C.M., Missmer S.A. (2020). Endometriosis. N. Engl. J. Med..

[2] Tanbo T., Fedorcsak P. (2017). Endometriosis-associated infertility: Aspects of pathophysiological mechanisms and treatment options. Acta Obstet. Gynecol. Scand..

[3] Gruber T.M., Mechsner S. (2021). Pathogenesis of Endometriosis: The Origin of Pain and Subfertility. Cells.

[4] Li W.N., Wu M.H., Tsai S.J. (2021). Hypoxia and Reproductive Health: The role of hypoxia in the development and progression of endometriosis. Reproduction.

[5] Hohmann T., Dehghani F. (2019). The Cytoskeleton-A Complex Interacting Meshwork. Cells.

[6] Adammek M., Greve B., Kassens N., Schneider C., Bruggemann K., Schuring A.N., Starzinski-Powitz A., Kiesel L., Gotte M. (2013). MicroRNA miR-145 inhibits proliferation, invasiveness, and stem cell phenotype of an in vitro endometriosis model by targeting multiple cytoskeletal elements and pluripotency factors. Fertil. Steril..

[7] Kwiatkowski S., Seliga A.K., Vertommen D., Terreri M., Ishikawa T., Grabowska I., Tiebe M., Teleman A.A., Jagielski A.K., Veiga-da-Cunha M. (2018). SETD3 protein is the actin-specific histidine N-methyltransferase. eLife.

[8] Terman J.R., Kashina A. (2013). Post-translational modification and regulation of actin. Curr. Opin. Cell Biol..

[9] Johnson P., Harris C.I., Perry S.V. (1967). 3-methylhistidine in actin and other muscle proteins. Biochem. J..

[10] Asatoor A.M., Armstrong M.D. (1967). 3-methylhistidine, a component of actin. Biochem. Biophys. Res. Commun..

[11] Wilkinson A.W., Diep J., Dai S., Liu S., Ooi Y.S., Song D., Li T.M., Horton J.R., Zhang X., Liu C. (2019). SETD3 is an actin histidine methyltransferase that prevents primary dystocia. Nature.

[12] Tiebe M., Lutz M., Levy D., Teleman A.A. (2018). Phenotypic characterization of SETD3 knockout Drosophila. PLoS ONE.

[13] Cohn O., Feldman M., Weil L., Kublanovsky M., Levy D. (2016). Chromatin associated SETD3 negatively regulates VEGF expression. Sci. Rep..

[14] Abaev-Schneiderman E., Admoni-Elisha L., Levy D. (2019). SETD3 is a positive regulator of DNA-damage-induced apoptosis. Cell Death Dis..

[15] Raychaudhuri P., Park H.J. (2011). FoxM1: A master regulator of tumor metastasis. Cancer Res..

[16] Zona S., Bella L., Burton M.J., Nestal de Moraes G., Lam E.W. (2014). FOXM1: An emerging master regulator of DNA damage response and genotoxic agent resistance. Biochim. Biophys. Acta.

[17] Engqvist H., Parris T.Z., Kovacs A., Ronnerman E.W., Sundfeldt K., Karlsson P., Helou K. (2020). Validation of Novel Prognostic Biomarkers for Early-Stage Clear-Cell, Endometrioid and Mucinous Ovarian Carcinomas Using Immunohistochemistry. Front. Oncol..

[18] Xu L., Wang P., Feng X., Tang J., Li L., Zheng X., Zhang J., Hu Y., Lan T., Yuan K. (2019). SETD3 is regulated by a couple of microRNAs and plays opposing roles in proliferation and metastasis of hepatocellular carcinoma. Clin. Sci..

[19] Pires-Luis A.S., Vieira-Coimbra M., Vieira F.Q., Costa-Pinheiro P., Silva-Santos R., Dias P.C., Antunes L., Lobo F., Oliveira J., Goncalves C.S. (2015). Expression of histone methyltransferases as novel biomarkers for renal cell tumor diagnosis and prognostication. Epigenetics.

[20] Cheng X., Hao Y., Shu W., Zhao M., Zhao C., Wu Y., Peng X., Yao P., Xiao D., Qing G. (2017). Cell cycle-dependent degradation of the methyltransferase SETD3 attenuates cell proliferation and liver tumorigenesis. J. Biol. Chem..

[21] Gabriel M., Fey V., Heinosalo T., Adhikari P., Rytkonen K., Komulainen T., Huhtinen K., Laajala T.D., Siitari H., Virkki A. (2020). A relational database to identify differentially expressed genes in the endometrium and endometriosis lesions. Sci. Data.

[22] Luo X., Cheng W., Wang S., Chen Z., Tan J. (2018). Autophagy Suppresses Invasiveness of Endometrial Cells through Reduction of Fascin-1. BioMed Res. Int..

[23] Ibrahim M.G., Delarue E., Abesadze E., Haas M., Sehouli J., Chiantera V., Mechsner S. (2017). Abdominal Wall Endometriosis: Myofibroblasts as a Possible Evidence of Metaplasia: A Case Report. Gynecol. Obstet. Investig..

[24] Schuler L. (1985). Selection for fertility in mice—The selection plateau and how to overcome it. Theor. Appl. Genet..

[25] Dietl G., Langhammer M., Renne U. (2004). Model simulations for genetic random drift in the outbred strain Fzt:DU. Arch. Anim. Breed..

[26] Langhammer M., Michaelis M., Hartmann M.F., Wudy S.A., Sobczak A., Nurnberg G., Reinsch N., Schon J., Weitzel J.M. (2017). Reproductive performance primarily depends on the female genotype in a two-factorial breeding experiment using high-fertility mouse lines. Reproduction.

[27] Ludwig C.L.M., Bohleber S., Rebl A., Wirth E.K., Venuto M.T., Langhammer M., Schweizer U., Weitzel J.M., Michaelis M. (2022). Endocrine and molecular factors of increased female reproductive performance in the Dummerstorf high-fertility mouse line FL1. J. Mol. Endocrinol..

[28] Langhammer M., Wytrwat E., Michaelis M., Schon J., Tuchscherer A., Reinsch N., Weitzel J.M. (2021). Two mouse lines selected for large litter size display different lifetime fecundities. Reproduction.

[29] Shu W.J., Du H.N. (2021). The methyltransferase SETD3-mediated histidine methylation: Biological functions and potential implications in cancers. Biochim. Biophys. Acta Rev. Cancer.

[30] Hassan N., Rutsch N., Gyorffy B., Espinoza-Sanchez N.A., Gotte M. (2020). SETD3 acts as a prognostic marker in breast cancer patients and modulates the viability and invasion of breast cancer cells. Sci. Rep..

[31] Kim D.W., Kim K.B., Kim J.Y., Seo S.B. (2011). Characterization of a novel histone H3K36 methyltransferase setd3 in zebrafish. Biosci. Biotechnol. Biochem..

[32] Dmowski W.P., Ding J., Shen J., Rana N., Fernandez B.B., Braun D.P. (2001). Apoptosis in endometrial glandular and stromal cells in women with and without endometriosis. Hum. Reprod..

[33] Depalo R., Cavallini A., Lorusso F., Bassi E., Totaro I., Marzullo A., Bettocchi S., Selvaggi L. (2009). Apoptosis in normal ovaries of women with and without endometriosis. Reprod. BioMed. Online.

[34] Zeitvogel A., Baumann R., Starzinski-Powitz A. (2001). Identification of an invasive, N-cadherin-expressing epithelial cell type in endometriosis using a new cell culture model. Am. J. Pathol..

[35] Ramirez Williams L., Brüggemann K., Hubert M., Achmad N., Kiesel L., Schäfer S.D., Greve B., Götte M. (2019). Γ-Secretase inhibition affects viability, apoptosis, and the stem cell phenotype of endometriotic cells. Acta Obstet. Gynecol. Scand..

[36] Cora M.C., Kooistra L., Travlos G. (2015). Vaginal Cytology of the Laboratory Rat and Mouse: Review and Criteria for the Staging of the Estrous Cycle Using Stained Vaginal Smears. Toxicol. Pathol..

[37] Byers S.L., Wiles M.V., Dunn S.L., Taft R.A. (2012). Mouse estrous cycle identification tool and images. PLoS ONE.

[38] Pfaffl M.W. (2001). A new mathematical model for relative quantification in real-time RT-PCR. Nucleic Acids Res..

[39] Pfaffl M.W., Horgan G.W., Dempfle L. (2002). Relative expression software tool (REST) for group-wise comparison and statistical analysis of relative expression results in real-time PCR. Nucleic Acids Res..

